# Adult Gross Motor Learning and Sleep: Is There a Mutual Benefit?

**DOI:** 10.1155/2018/3076986

**Published:** 2018-08-13

**Authors:** Monica Christova, Hannes Aftenberger, Raffaele Nardone, Eugen Gallasch

**Affiliations:** ^1^Institute of Physiotherapy, University of Applied Sciences FH-Joanneum, Graz, Austria; ^2^Otto Loewi Research Center, Physiology Section, Medical University of Graz, Graz, Austria; ^3^Department of Neurology, Franz Tappeiner Hospital, Merano, Italy; ^4^Department of Neurology, Christian Doppler Clinic, Paracelsus Medical University, Salzburg, Austria

## Abstract

Posttraining consolidation, also known as offline learning, refers to neuroplastic processes and systemic reorganization by which newly acquired skills are converted from an initially transient state into a more permanent state. An extensive amount of research on cognitive and fine motor tasks has shown that sleep is able to enhance these processes, resulting in more stable declarative and procedural memory traces. On the other hand, limited evidence exists concerning the relationship between sleep and learning of gross motor skills. We are particularly interested in this relationship with the learning of gross motor skills in adulthood, such as in the case of sports, performing arts, devised experimental tasks, and rehabilitation practice. Thus, the present review focuses on sleep and gross motor learning (GML) in adults. The literature on the impact of sleep on GML, the consequences of sleep deprivation, and the influence of GML on sleep architecture were evaluated for this review. While sleep has proven to be beneficial for most gross motor tasks, sleep deprivation in turn has not always resulted in performance decay. Furthermore, correlations between motor performance and sleep parameters have been found. These results are of potential importance for integrating sleep in physiotherapeutic interventions, especially for patients with impaired gross motor functions.

## 1. Introduction

Several human behaviors such as playing sports, playing music, and handcrafting are composed of unique combinations of gross and fine motor skills. Perfect execution of such highly coordinated tasks involves complex operations within the sensory and motor control structures [1, 2] including learning over a long period of time [3]. Learning commonly starts with initial task acquisition and results in reaching proficiency and stabilization of the learned information for further recall. Within the learning process, two phases can commonly be discriminated: encoding and consolidation. While encoding refers to the initial performance improvement occurring during practice (online learning), consolidation refers to the stabilization of memories during a period after practice [4, 5]. After consolidation, an additional performance improvement may occur even in the absence of further practice, an effect denoted as offline gain or offline learning [6]. Depending on the specificity of the task, such offline gains can occur during wakefulness but can also occur during diurnal or nocturnal sleep [7–9].

An extensive amount of literature has provided evidence that sleep plays an active role in the consolidation of memories [10–12]. The majority of the studies have addressed explicit memory and the role of the hippocampus in the formation of long-term memory. For example, by using a word list remembering task, consolidation was shown to take place during slow-wave sleep (SWS) rather than during rapid eye movement (REM) sleep [13]. In the case of consolidation of implicit memory, most studies focus on fine motor skills, such as serial reaction time tasks and sequential finger tapping tasks [8, 14–16]. Conclusions derived from research on cognitive or fine motor tasks do not generalize to gross and more complex motor skills [17]. The literature addressing gross motor skills primarily focuses on motor development in childhood and infancy, and to date, there is little knowledge about the role of sleep in adult gross motor learning (GML).

This review focuses on sleep and learning of novel gross motor skills in adults. Acquiring gross motor skills (e.g., dancing, playing a musical instrument, and golfing) often requires stepwise learning under the supervision of a demonstrator and, therefore, is less comparable to the learning of repetitive tasks such as finger tapping in front of a computer screen. Gross movements involve larger body segments and require more complex muscle synergies including postural stabilization and anticipatory adjustment [18]. Therefore, cortical and subcortical structures are likely involved in the encoding and consolidation of such skills [19]. Furthermore, training of gross motor skills often involves large muscle groups, which may lead to muscle fatigue and physical exhaustion [20]. It is therefore conceivable that GML also influences sleep duration and sleep architecture similarly to athletic exercise [21], and the question arises whether there is a mutual relationship between sleep and GML. More detailed knowledge on the relationship between sleep and GLM could be of relevance for physical therapy, as well as for the treatment of motor disabilities after stroke or brain tumor surgery.

Thus, in the present review, three aspects are highlighted to reveal possible relationships between GML and sleep. The first aspect focuses on the impact of sleep on skill consolidation compared to wakefulness. Here, bimanual tasks, dancing, inverse steering bicycling, or cascade juggling in combination with diurnal/nocturnal sleep are addressed. The second aspect focuses on the opposite direction, namely, whether GML can affect sleep architecture. Specifically, the effect of sports (trampoline, snakeboard) on REM and SWS is described in order to examine a possible correlation between the learning process and sleep parameters. Finally, the third aspect focuses on the impact of sleep deprivation on GML and memory consolidation. To this end, we review studies with a stepwise decrease in sleep duration as an experimental approach, mainly in the field of sports and virtual reality training.

## 2. Methods

The present work is a comprehensive review of computerized medical literature databases and searches. The MEDLINE database, accessed by PubMed electronic databases, was searched using the following free terms and medical subject headings combined in multiple search strategies: “gross motor learning/memory/skill,” “complex motor skill,” “motor adaptation,” “sleep,” “offline learning,” “consolidation,” and “deprivation.” The search was limited to studies written in English. Studies including infants and children (up 14 years old) were excluded. No other exclusion criteria were applied, in particular regarding the number of participants, presence of a placebo group, or outcome measures. Full-text articles were retrieved for the selected titles, and reference lists of the retrieved articles were screened for additional publications. Only original articles (excluding single case reports) reporting data on studies examining the relationship between GML and sleep were considered eligible for inclusion. Gross motor tasks are defined here as tasks involving at least three joints, uni- or bimanual, as well as whole-body movements. In advance of this review, an introductory chapter on memory formation and sleep as identified by research on cognitive and fine motor tasks, is provided.

## 3. Common Mechanisms in Memory Formation and Sleep

The process of memory formation involves two main phases: encoding and consolidation. The encoding phase is associated with hippocampal long-term potentiation (LTP) plasticity [22], which involves the formation of a new memory trace that is initially fragile and vulnerable to external influences. Second, in the consolidation phase, a fragile memory trace is transferred to more permanent long-term storage throughout the neocortex [23] for further recall during retrieval. Thus, the consolidation phase is also associated with systemic reorganization. The significance of the consolidation phase has been explored by means of pharmacological and electrophysiological interventions administered at different time windows after learning [24, 25].

The perception and processing of information during encoding and retrieval requires the awake and active state of the brain. In contrast, skill consolidation takes place in the absence of attention and during sleep. There is assumed to be less interference from other stimuli during sleep, which protects the stabilization of a newly created memory trace [26–28]. In addition to this protective role of sleep in a passive manner, a reactivation of memory representations in hippocampal and nonhippocampal areas via synaptic plasticity mechanisms has been demonstrated in animal models [29] and in human studies [30, 31] during the different sleep phases, predominantly in SWS (for a review, see also [10]).

Two theoretical models have been proposed for these interactions between memory formation and sleep: the active system consolidation hypothesis and the synaptic homeostasis hypothesis. The first model refers to the dialog between the hippocampus and the neocortex, which is associated with learning during wakefulness and reactivation during non-REM (NREM) sleep [32]. This reactivation ensures the redistribution of new information within cortical networks via strengthening of synaptic connections [33]. According to the synaptic homeostasis hypothesis [34], strengthening of synaptic connections occurs during encoding in wakefulness. During subsequent SWS, synaptic strengthening becomes renormalized, thus removing irrelevant and less integrated information and restoring the synaptic capacity for new learning.

Increased protein synthesis, as required for synaptic strengthening, was first found during NREM sleep [35]. Specifically, the stage of NREM sleep is proposed as the period in which short-lasting LTP is converted to longer-lasting LTP involving new protein synthesis [36]. In the absence of protein synthesis, short-lasting LTP will fade out after some hours [37]. To this end, sleep has been reported to elevate cortical messenger RNA levels of genes associated with protein synthesis [38, 39], which are critical for strengthening existing synapses and building new ones (for a review, see [24]). In addition, different processes of synaptic reorganization occur during NREM and REM sleep, as summarized in the review by Gorgoni et al. [40]. Finally, electrophysiological markers within sleep stages NREM2, SWS (stages NREM3 and NREM4 according to the classification of Kales and Rechtschaffen [41]), and REM have also been related to the induction of LTP-like plasticity in the context of memory consolidation.

Sleep stage NREM2 is characterized by the presence of sleep spindles and K-complexes, and here the sleep spindles play a functional role in memory consolidation. Sleep spindles, defined as bursts at the sigma frequency range between 11 and 16 Hz and lasting up to 3 sec [42–44], are generated within the thalamic reticular nucleus. Spindle activity causes Ca^2^ influx at the dendrites of pyramidal neurons and triggers a cascade of molecular processes, which lead to gene expression and protein synthesis necessary for LTP of the postsynaptic membrane of neocortical synapses [45, 46]. Furthermore, LTP at excitatory synapses is linked to a growth of synaptic spines [47]. An increase in dendritic spines after motor learning in mice was shown to be promoted by NREM2 sleep [29]. Positive correlations have been found between spindle duration and density with offline learning [48, 49] but not with nonspecific motor activity [50]. Specifically, increased spindle activity was found after visuomotor tasks [51] and after finger motor sequences [52]. Also using a motor finger sequence task but with experimental cuing with odor during NREM2 sleep, Laventure et al. [53] demonstrated that sleep spindles in particular contribute to the consolidation of motor sequence memories. These findings suggest the importance of sleep spindle activity for the strengthening of motor memory traces, promoted by functional and structural plasticity.

The SWS stage is characterized by the prevalence of slow oscillations, which represent the neuronal membrane potential oscillations that are expressed in the electroencephalogram (EEG) as slow-wave activity (SWA) within the 0.5–4 Hz frequency band [54, 55]. The slow oscillations are of thalamocortical origin and comprise periods of membrane depolarization (sustained firing) alternated with periods of membrane hyperpolarization (neuronal silence). While the activity during the depolarization phases has been attributed to corticocortical glutamatergic synaptic connections [56], which reflect an excitatory/inhibitory balance, the hyperpolarization phases have been related to intracellular mechanisms suppressing neuronal excitability [57]. The significance of slow oscillations in the formation of motor memory was demonstrated during training of a visuomotor adaptation task [58] that increased the SWA, which was correlated with improved task performance after sleep. On the other hand, slow-wave deprivation impaired the sleep-related consolidation of a visuomotor adaptation task [59], whereas boosting the slow oscillations with low-frequency transcranial alternating current stimulation facilitated the consolidation of declarative memory [60]. The role of slow waves in memory consolidation has been attributed both to synaptic depression and synaptic potentiation mechanisms (for a review, see [61]).

The REM sleep stage, characterized by desynchronized EEG activity, is sensitive to the induction of synaptic plasticity changes. The waves of excitation (ponto-geniculo-occipital, PGO waves) during REM sleep were first described in the rat brainstem [62]. These waves project to the hippocampus and the amygdala [63] and show increased intensity and density after intensive learning, which correlates with task improvement [64]. These waves have also been proposed as regulators of synaptic plasticity, since they are comprised of waves of glutamate terminating on forebrain areas [65]. In addition to PGO waves, factors such as theta activity, increased acetylcholine levels, and increased transcription of plasticity-related genes during REM sleep [66] contribute to the induction of bidirectional plasticity (LTP/LTD). Bidirectional plasticity supports memory-associated synaptic remodeling in the hippocampus [67]. In addition, REM sleep has been demonstrated to selectively eliminate and maintain the postsynaptic dendritic spines of layer 5 pyramidal neurons in the mouse motor cortex during motor learning and memory consolidation [68]. Human imaging studies [69] have shown increased post training activation during REM sleep in the brain areas involved in task acquisition.

## 4. Effects of Sleep on Gross Motor Learning

The effect of day/night sleep on GML was examined with uni- and bimanual motor tasks, as well as with whole-body movements in healthy volunteers. In the study of Kempler and Richmond [70], the task consisted of bimanual movements, involving sequential combinations of three positions with both arms simultaneously. This task, performed by 70 adults, was initially practiced for 6 min with video assistance and then retested, whereby the number of accurate cycles was calculated. Participants showed a higher number of accurate cycles of the task at retest after nocturnal sleep but did not exhibit a significant change after wakefulness. Another study, implementing bimanual movements [71], examined the influence of night sleep in adaptive skill learning. Right-handed university students played a shooter video game, which requires fast responses to changing visual and auditory stimuli. In this task, the players simultaneously manipulated the keyboard with the left hand and the mouse with the right hand. A training period of 28 min, preceded by a baseline score evaluation, was performed in the morning or in the evening. Posttraining tests were carried out immediately after training, or 12 or 24 hours after training in separate groups. Performance improved along with training and then deteriorated after 12 hours wakefulness. However, performance recovered and stabilized after night sleep. Sleep-dependent learning gains were also reported by Kuriyama et al. [72] who demonstrated not only that performance on a complex nine-element bimanual finger taping task could benefit from night sleep (28.9% improvement) but also that these gains correlated with task complexity and coordination. The results were compared to more simple tasks, which showed 17–20% overnight improvement. Interestingly, the maximum benefit was observed for the most difficult skills, which were unable to be mastered prior to sleep.

Performance gains after nocturnal sleep have also been demonstrated at unimanual tasks. Malangre and Blischke [73] and Malangre et al. [74] employed a pegboard task on an electronic board where a sequence of gross reaching movements including the joints of the wrist, elbow, and shoulder were performed with the nondominant hand in the horizontal plane. One group practiced in the morning, the second in the evening. Retests were carried out 15 min after acquisition in order to control the early retention as well as after 12 and 24 hours [73]. Mean execution time along all retests was reduced after nocturnal sleep but not after the wake periods.

Sleep-related effects were also examined with coordination movements involving the whole body. Long complex dance choreography was implemented on a PlayStation 2 Game Dance Stage [75]. Using constant visual feedback, young male volunteers learned a dance consisting of a set of sequential movements in the evening or in the morning. Twelve and 24 hours later, they were retested on the same choreography in order to assess sequence-specific learning but were also tested on a new set of movements in order to examine the transfer from a newly acquired skill to a novel similar task. Sleep resulted in improved performance when the same dance was retested; however, the performance of the new set of dance movements was not improved by sleep.

In addition to nocturnal sleep the effectiveness of diurnal sleep on GML has also been examined. Before and after a 2-hour day nap, during which NREM and REM sleep were controlled with polysomnographic recordings, young female subjects performed a highly coordinated three-ball cascade juggling for 15 min [76]. Juggling performance significantly improved at retest in the nap group but not in the awake group. Moreover, these performance gains were further retained on the following day [77]. The effect of a 2-hour midday nap on a complex posturolocomotor task (learning to ride an inverse steering bicycle) was investigated in another recent study [78]. The authors implemented straight-line or slalom bike riding and, in contrast to the previous studies, there was no benefit from the midday nap. Moreover, a significant decrease in accuracy at slalom and at straight-line riding was found after the nap but also after wakefulness. The performance decrease was negatively related to the sleep parameters (REM duration and spindle activity). These findings were attributed to the need to forget more recently acquired interfering tasks in order to protect more relevant skills that are needed daily.

A “multitask research strategy” was used by Blischke et al. [79] to investigate the effect of nocturnal sleep on learning a set of different task domains, including finger tapping tasks, pursuit tracking and countermovement jump, where subjects were required to produce a vertical force impulse of 60% of the individual maximum. Whereas performance of small finger movements (sequential finger tapping) was improved after sleep, gross body movements (vertical jump) remained stable across the sleeping period. These results indicate a differential effect of nocturnal sleep on small and gross motor learning.

In contrast, a beneficial effect of overnight sleep on learning a novel walking task was found by Al-Sharman and Siengsukon [80]. This task consisted of walking along an irregular elliptical path approximately 30 m long and 0.5 m wide while performing a mental cognitive task (counting backwards) in order to approximate walking in a natural environment. The task required whole-body coordination and adaptation to environmental stimuli. Improved step length and reduced time were found at retest after 12 hours including 7 hours of sleep but not after 12 hours awake. Importantly, a correlation between sleep quality and offline learning was reported.

## 5. Factors Influencing the Effect of Sleep on GML

While in the majority of these studies sleep enhanced gross motor performance [72–74, 76, 77, 80], others have reported stabilization without further improvement [71, 79] or even performance deterioration [78]. Factors such as type of motor task, training specificity, presleep performance level and complexity can be causes for such inconsistencies. Complex explicit tasks with high cognitive demands such as sequential bilateral arm or manual movements, cascade juggling, or walking with counting generally benefit from sleep. Moreover, performance gains correlate positively with task complexity [72]. One reason could be that less complex skills, being easier to master, reach a ceiling effect before sleep. Another reason could be that greater cognitive efforts induce fatigue, which can be successfully restored in sleep. The importance of task complexity was emphasized in a recent study [81] wherein the sleep-related improvements were absent with shorter sequences and more regular movement patterns.

Studies employing whole-body postural tasks, which mainly involve implicit learning strategies (vertical jump, inverse bicycling), have reported an absence of effects or even decreased performance accuracy at retest. Broadly, implicitly acquired movements such as vertical jump and inverse bicycling [78], which predominantly involve procedural memory, do not appear to benefit from sleep for memory consolidation. A similar differential effect of sleep, whether a task is explicit or implicit, was reported by Robertson et al. [6] for nongross motor sequence learning. In addition, at such whole-body postural tasks, participants are unlikely to reach asymptotic performance after a short training period, which can limit the postsleep performance gains, as shown by Hauptmann et al. [82]. Finally, the possibility that irrelevant movement patterns, such as riding a bicycle in an inverse direction, tend to be removed during sleep in order to selectively enhance the memories for activities pertinent to daily life cannot be excluded, which is in accordance to the synaptic homeostasis hypothesis [34]. Additionally, transfer of new dance choreography movements was not promoted by sleep [75], which suggests that adaptation to new settings might occur independently of sleep. Perhaps representations that were not particularly engaged in the learning preceding sleep and therefore not involved in the formation of the movement schemata [83], cannot be influenced by the SWS downscaling and therefore remain unaffected by sleep.

Further important factors concerning sleep-related learning are the time-of-day effect, sleep duration, and sleep environment. Performing the trainings/retests at opposite times of day (morning/evening for the wake group versus evening/morning for the sleep group) as in the study of Al-Sharman and Siengsukon [80] may account for different offline gains because of endogenous circadian influences [84]. An equitable testing can be achieved by using a study design with 24-hour delay as done in other studies [70, 73]. In most of the GML studies, the sleeping duration was between 6 and 8 hours at night. Commonly, the participants did not spend the night in a sleep lab; therefore, recording of sleep quality was carried out using sleepiness scales and questionnaires [70, 74], actigraphy [80], or self-report [75]. Only sleeping overnight in a sleep lab ensures objective assessment of sleep architecture, which enables the determination of possible correlations between the sleep parameters and learning scores. On the other hand, an overnight stay in an unfamiliar environment may influence sleep quality, a problem that can be solved by providing a baseline night for familiarization. In some studies, the quality of diurnal sleep has been controlled with polysomnography [76–78], which enables comparisons between the sleep and learning parameters. Despite the fact that a daytime nap and a full night's sleep have different physiological characteristics, they are both able to induce behavioral gains in GML. This observation was also demonstrated using the same task (juggling) in the studies of Morita et al. [76, 77].

Similar results, in which improvements occurred after a whole night's sleep [15, 75] but also after a short nap [8, 85], have been reported for simple motor sequence tasks. A direct comparison between diurnal and nocturnal sleep [86] revealed lower spindle density but higher spindle activity and amplitude in daytime naps compared to those in night sleep. Furthermore, the same study showed that daytime naps protected procedural memories (mirror tracing task) from deterioration, whereas a full night's sleep improved performance. Since the presence of both REM and SWS phases are crucial for evolving LTP changes and memory consolidation, it is of interest to examine whether longer sleep is associated with higher GML gains. Additionally, defining the minimum/effective duration of diurnal sleep could be of importance when scheduling practice-rest in rehabilitation sessions, for example.

Age can influence sleep-related motor learning gains, as shown with simple motor tasks. For example, postsleep consolidation in older adults was found to be dependent on the movement kinematic [87]: while a finger sequence task failed to show sleep-related gains, a kinematically adapted gross motor whole-hand task showed sleep-dependent consolidation. In addition, studies using less complex tasks such as finger sequences [88] and mirror tracking [89] also reported lack of sleep-dependent consolidation (for a review, see also [90]). The majority of the studies on GML have focused on sleep-induced performance gains in young adults. Only the study of Al-Sharman and Siengsukon [91] verified these effects in the elderly, showing decreased walking time and increased accuracy after sleep in middle-aged and older individuals.

Older individuals also show a changed sleep architecture characterized by a reduction in total sleep time, REM, SWS [92–94], and sleep spindles [95], which have been shown to be important for memory consolidation and performance improvement [48]. However, such aging-related changes cannot explain why older adults can benefit from sleep after GML but not after learning fine motor tasks, as shown by Gudberg et al. [87]. Apparently, gross motor movements are more complex, thus requiring activation of larger brain networks, which appears to be a higher demand on motor-controlling structures in the elderly than in young individuals. This higher demand could still be accomplished by sleep even at reduced sleep efficiency. Alternatively, improvement in fine motor tasks could be limited in older adults as a result of aging [96]. Future research, including analysis of resting state networks, could be helpful to examine and compare sleep-related gross and fine ML consolidation in different age groups.

## 6. Effect of Sleep Deprivation on GML

Sleep is a state of reduced energy demands at the cellular and network level and is thus essential for maintaining behavioral and cognitive capabilities [97]. Sleep deficiency causes deterioration of motor and neurocognitive performance and involves changes at several systematic levels, for example, decreased physical performance, increased mental fatigue, changes in metabolism and endocrine functions, pain perception, and cognitive and emotional changes (for a review, see [98, 99]). In the brain, lack of sleep has an impact on neurotransmitter release [100, 101], which results in a decreased capacity to learn, store, and retrieve the learned material. Such decreased learning capacity might be partially explained by alterations in use-dependent synaptic plasticity.

Molecular and electrophysiological studies show that sleep loss inhibits hippocampal LTP and facilitates LTD induction [102, 103]. Additionally, prolonged wakefulness has been associated with net synaptic potentiation, whereas sleep preserves the overall balance of synaptic strength [104]. In support of these findings, transcranial magnetic stimulation (TMS) studies on humans have demonstrated that sleep deprivation increases cortical excitability [105] and decreases intracortical inhibition [106]. If LTP and cortical excitability are increased after sleep deprivation, then further learning-induced LTP would be less effective, which might be a reason for the observed learning and memory decline. Furthermore, sleep deprivation may impair memory consolidation by reducing the synthesis of proteins needed to support synaptic plasticity [107].

The effect of sleep loss on motor learning has been studied in healthy subjects using experimentally induced sleep deprivation. Sleep deprivation is commonly defined as a sleep time of less than 4 hours per 24 hours [108], although different durations have been reported. In one of the first works dedicated to the impact of sleep deprivation on gross motor performance, Holland [109] investigated the effect of one night of wakefulness on a jump and manipulation task in male college students. The experimentally imposed sleeplessness did not affect speed or accuracy of both discrete short-term tasks but did decrease long-term physical performance, as measured by a bicycle work test. Performance decline after sleep deprivation has been demonstrated in athletes in a variety of exercises (for a review, see [110]). While psychomotor tasks such as reaction time were diminished after sleep loss, gross motor functions remained unaffected [111]. Tasks involving longer-lasting physical efforts or higher cognitive demands tend to be more sensitive to sleep deficits.

Further evidence for the impact of sleep deprivation on GM training comes from studies examining bimanual dexterity in medical residents. Lehmann et al. [108] used a virtual surgery stimulator task, which involved both arms. Surgical residents and medical students were initially trained in tasks lasting between 20 and 30 min for 5 days in order to reach comparable skill levels. The subsequently reduced sleep duration (1.6–3.8 hours) did not influence motor or cognitive performance. Comparable findings were reported by DeMaria et al. [112] after learning of laparoscopic skills. In contrast, Eastridge et al. [113] and Taffinder et al. [114] found an increased number of errors and time to complete all tasks on simulated laparoscopy after sleep deprivation. The heterogeneity of these findings can be explained by the variability in sleep and task duration and the different proficiency levels of the residents in the different studies. Furthermore, the participants in these studies were to some extent acquainted with the laparoscopic tasks but also with the limited and irregular amount of sleep, thus being less vulnerable to both task acquisition and sleep loss. Therefore, these results cannot be directly considered for naive subjects or patients.

The effect of sleep deprivation on motor learning has also been studied in patients with sleep disorders. Sleep disorders such as insomnia, narcolepsy, or obstructive sleep apnea (OSA), which are characterized by elevated arousal and abnormal sleep architecture, result in a reduced capacity for consolidating explicit motor sequences and motor adaptation skills (for a review, see Cellini [115]).

## 7. Effect of GML on Sleep Architecture

Sleep following learning of gross motor skills not only facilitates memory consolidation but may also influence sleep homeostasis. For example, motor learning in rats induced a local increase in SWA in the cortical region directly involved in the motor task [116], and the SWA increase correlated positively with performance improvement. In humans, evidence from earlier studies has demonstrated the influence of aerobic but not anaerobic exercise on sleep variables [117–119] with specific alterations in NREM2 and SWS stages, which reflect increased activity of the metabolic recovery processes after extensive motor activity. In later studies, the effect of GLM on sleep variables has been shown primarily with whole-body coordination movements.

The effect of learning a new complex sport activity (trampolining) on REM sleep was investigated by Buchegger et al. [120] and compared to the effect of learning other control anaerobic tasks in a 13-week program once a week for 2 hours. Only the trampoliners showed a significant increase in subsequent REM sleep, a result that probably reflects the motor complexity of the task. Implementing another procedural whole-body coordination task, Erlacher and Schredl [121] studied the effect of gross motor learning on sleep in a balanced within-subject design. Subjects learned either snakeboard riding for 2 hours or took part in a control ergometry task for the same period. However, no difference was found between the experimental and control condition in sleep, REM parameters, or subjective sleep rating. Using a combination of simple procedural tasks (pursuit rotor, simple tracing, operation task, and ball and cup) Fogel and Smith [48] found that sleep spindle density at stage NREM2 was positively related to the overall task improvement without involvement of REM sleep mechanisms. Since gross motor learning was represented by only one of the tasks in this study (ball and cup), it cannot be concluded whether this task alone would have produced the same effect. However, in another study by Milner et al. [122], the effect of the “ball and cup” task solely on a 20 min daytime nap was investigated in habitual and nonhabitual nappers. Interestingly, the number of sleep spindles and sigma power (13.5–15 Hz) in stage NREM2 predicted the task performance following the nap but only in the habitual nappers.

Two studies, primarily investigating the effect of diurnal sleep on GML, also examined the influence of the task on REM/NREM sleep without, however, comparison to a control task. In the first study, cascade juggling for 15 min showed an effect on day nap [76], and alterations in EEG spectral power relative to that in a baseline nap were observed during NREM sleep. Specifically, there was an increase in spectral power in the band related to slow waves (0.5–1.5 Hz, “delta band”) and in the band related to sleep spindles (11.5–15.5 Hz, “sigma band”). These increases were correlated with improved motor performance. Similarly, higher sleep spindle activity and longer REM durations were found in the second study, after learning a novel complex gross motor task (riding an inverse steering bicycle) by Hoedlmoser et al. [78], although the authors observed a negative correlation with task improvement.

The variable findings concerning the effect of GML on sleep parameters and the correlation between performance gains and sleep architecture could be due to the methodological differences and the level of task difficulty. A night prior to the study for adaptation and control of the first-night effect [123] was not provided in these studies. Apparently, individual sleep habits should also be considered, especially in studies where daytime sleep/nap is examined. Additionally, the tested participants in some studies had a background in sports, while in other studies, the participants did not, which could account for the differences in responsiveness to the task nature and complexity. Nagai et al. [124] demonstrated the importance of task complexity for sleep consolidation by using Fos expression to evaluate neuronal activation in mice. Their results showed that complex but not simple training engaged the motor cortex and the hippocampus to a greater extent and induced a longer sleep duration, which was correlated with greater performance.

Overall, NREM sleep variables were mostly influenced by GML, probably via synaptic potentiation mechanisms occurring after learning. This observation has also been supported by animal studies showing that changes in SWA are driven by synaptic potentiation [116] after learning a task involving an increase in dendritic branching at layers II, III, and V [125] and an enhancement in the strength of horizontal intracortical connections [126]. An increase in SWA was also reported after application of other LTP induction interventions such as 5 Hz repetitive TMS of M1 [127] or paired associative stimulation [128].

## 8. Importance for Rehabilitation Practice

The motor rehabilitation process, particularly that for physio- and occupational therapy, involves relearning of lost skills or learning of compensatory/substitution tasks. These tasks are commonly gross motor actions, which consist of motor sequences, complex coordination patterns, and motor adaptations, for example walking with prosthesis, performing daily life activities with a paretic hand, or navigation of a wheelchair by patients with spinal cord injuries. The acquisition of such tasks is associated with the development of new motor strategies and large reorganizational map changes, which takes place over days and weeks. Thus, a proper scheduling of single therapy sessions along with rest/sleep periods is relevant for the success of the therapy. Furthermore, the effectiveness of multiple naps for motor recovery over a longer time is of interest. The optimal integration of sleep in rehabilitation practice and maximization of the effect of sleep is also of particular importance for neurological conditions, in which gross motor functions are affected, such as in patients with neurological and neuropsychological disorders like cerebral palsy, Parkinson's disease, autism, and motor apraxia or in children with developmental disorders.

Considering the results from studies on healthy individuals, it could be of potential clinical benefit to incorporate, for example, a diurnal sleep of at least 90–100 min in order to increase the likelihood of the REM and NREM phases. Furthermore, since current evidence has shown that acquiring a movement set related to the learned one but containing new elements was not promoted by sleep [75], it may be advantageous to introduce a task before sleep and practice it in the same form after sleep. Considering that most neurological disorders also involve specific structural or functional changes within the learning-related brain structures, often together with cognitive changes, direct evidence coming from studies on patients is critical. The effect of sleep on motor learning in a stroke population has already been examined and summarized in previous reviews [129, 130]. Stroke victims, in contrast to healthy age-matched controls, have been reported to benefit from sleep when learning both implicit and explicit versions of discrete and continuous tasks. However, small finger tasks, performed with the less affected hand, were commonly utilized; therefore, direct conclusions regarding the consolidation of activities relevant to daily life cannot be drawn. Studies on stroke patients addressing the effect of sleep on learning whole-body or bimanual tasks in relation to lesion localization and poststroke time are warranted.

Sleep deficiency or changed sleep architecture is reported in several neurological conditions including multiple sclerosis [131, 132], Parkinson's disease [133], stroke [134], and traumatic brain injury [135, 136]. Further, associations between sleep characteristic and physical functions have been found in older veterans with different comorbidities [137]. Longer (>7.5 hours) or shorter (<6 hours) sleep time and fragmented sleep are significantly correlated with poor performance in daily life and instrumental daily life activities. Thus, defining sleep disorders, which may negatively influence motor performance in these risk groups, should be considered in physiotherapy. Improving sleep disturbances with pharmacological and nonpharmacological interventions may be assumed to in turn improve learning outcomes, as already demonstrated in patients with OSA [138]. Considering that learning complex GML tasks affects sleep architecture [76, 78], we anticipate a reciprocal effect: on one side, sleep disturbances might be normalized by motor performance; on the other side, normal sleep can stabilize and enhance GML gains.

## 9. Conclusions

GML and sleep interact via common synaptic plasticity mechanisms and, thus, can influence each other. Therefore, the question arises whether this influence is of a mutual benefit. In healthy adults, sleep can stabilize and improve the consolidation of more complex GM tasks, probably because of the higher cognitive/motor demands, which require more pronounced synaptic stabilization that takes place during sleep. The learning gains are demonstrated in both implicit and explicit tasks and predominantly in young adults. Only a few studies have addressed and shown these benefits in the elderly population as well. GML can be concluded to benefit from sleep. However, age- and gender-related sleep differences, as well as variability in individual sleep patterns, should be considered in future studies. In turn, training of GM tasks can influence sleep variables across all NREM sleep stages. Again, this effect is related to task complexity but also to sleep habits. Whether the performance-induced changes in sleep architecture can be interpreted as improved sleep quality remains speculative to conclude from the current findings. Moreover, a positive correlation between learning and sleep parameters was not shown in all studies.

Interestingly, some of the described effects are specific for GML and are thus in contrast to the findings on sleep and learning paradigms involving fine or simple motor skills. Therefore, further research, especially on neurological patients with impaired GM functions, is warranted. A better understanding of the interaction between sleep and GML may contribute to the optimization of therapeutic strategies by integrating sleep with physiotherapy for these patient groups.

## Additional Points

*Research Agenda.* (1) Characterisation of age-dependent effects on sleep-related gains using more complex gross motor tasks (e.g., juggling and dance choreography); (2) investigation of whether sleep promotes equal consolidation of daily life-relevant versus daily life-irrelevant tasks; (3) effects of sleep deprivation on GML performance involving whole-body movements; (4) comparison of sleep-related gains following learning of explicit versus implicit gross motor tasks; (5) comparison of sleep-related effects of gross versus fine motor learning in stroke patients; (6) effectiveness of daytime naps after motor therapy in physiotherapy on functional improvement in stroke patients; (7) relevance of lesion location and poststroke stage on sleep-related GM training; (8) testing of sleep-stage-dependent noninvasive brain stimulation for enhancing memory consolidation; and (9) influences of stimulating factors (music, sonification of movements, and augmented reality) on learning and sleep performance.
